# Comparison of the prognostic value of four different critical illness scores in patients with sepsis-induced coagulopathy

**DOI:** 10.1515/biol-2022-0659

**Published:** 2023-08-09

**Authors:** Chengli Wang, Li Ma, Wei Zhang

**Affiliations:** Department of Critical Care Medicine, 3201 Hospital, Hanzhong 723000, Shaanxi, China; Department of Microbiology, 3201 Hospital, No.783, Tian-han Road, Han-Tai District, Hanzhong 723000, Shaanxi, China

**Keywords:** sepsis-induced coagulopathy, prognosis, JAAM, ISTH, CDSS, CRUSADE4 score

## Abstract

In patients with sepsis-induced coagulopathy (SIC), the Chinese DIC scoring system (CDSS) of the Chinese Society of Thrombosis and Hemostasis score, the Japanese Association for Acute Medicine (JAAM) score, the International Society of Thrombosis and Hemostasis (ISTH), and the Can Rapid risk stratification of Unstable angina patients Suppress Adverse outcomes with Early implementation of the ACC/AHA Guidelines (CRUSADE) score were compared for their predictive significance (SIC). From August 2021 through August 2022, 92 SIC patients hospitalized in our hospital’s Department of Critical Care Medicine served as study participants. Groups of patients were created with a bad prognosis (*n* = 35) and a favorable prognosis (*n* = 57) 14 days following admission. Electronic medical records were used to compile patient information such as demographics (gender, age, and body mass index), medical history (hypertension, diabetes, chronic obstructive pulmonary disease, and chronic kidney disease), treatment (mechanical ventilation, APACHE II score at admission), and outcomes (results). All patients’ JAAM, CDSS, ISTH, and CRUSADE scores were recorded. The APACHE II scores of the group with a poor prognosis were noticeably (*p* < 0.05) higher upon admission than those of the group with a favorable prognosis. The poor prognosis group had higher JAAM, ISTH, CDSS, and CRUSADE scores than the good prognosis group (all *p* < 0.05). Partial coagulation indicators in fibrinogen, D-dimer, activated partial thromboplastin time, and prothrombin time were positively linked with JAAM, ISTH, CDSS, and CRUSADE (all *p* < 0.05). At admission, the JAAM, ISTH, CDSS, CRUSADE, and APACHE II scores were independently linked with SIC patients’ prognosis (all *p* < 0.05) in a multivariate logistic regression analysis. According to receiver operating characteristic analysis, the area under the curve for predicting the prognosis of SIC patients using the JAAM, ISTH, CDSS, and CRUSADE4 scores was 0.896, 0.870, 0.852, and 0.737, respectively, with 95% CI being 0.840–0.952, 0.805–0.936, 0.783–0.922 and 0.629–0.845, respectively (all *p* < 0.05). The prognosis of SIC patients may be predicted in part by their JAAM, ISTH, CDSS, and CRUSADE4 scores, with the CDSS score being the most accurate. This research provides important recommendations for improving the care of patients with SIC.

## 1 Introduction

Around 1.7 million individuals have sepsis each year in the United States, and about 265,000 die from sepsis [1,2,3]. An improper immune response to infection brings on organ failure, known as sepsis. Microcirculation disorders, tissue ischemia, hypoxia, and organ dysfunction are all caused by inhibiting the physiological fibrinolytic pathway, which is triggered by the inflammatory response in the early stage of sepsis [4,5,6]. This is a significant contributor to the poor prognosis of patients. Coagulopathy is a frequent sepsis complication that may occur in up to 32% of patients, dramatically increasing the death rate in sepsis patients [7].

Due to the severe condition of sepsis, there are many complications. At the same time, the coagulation function index is changed by non-coagulation dysfunction, which lacks specificity. Traditional coagulation function indicators have limited value in reflecting coagulation dysfunction in sepsis, so clinical data and multi-molecular markers need to be integrated to evaluate diagnosis and prognosis [8]. In 2017, the Japanese Association for Acute Medicine (JAAM) criteria, the Chinese DIC scoring system (CDSS) of the Chinese Society of Thrombosis and Hemostasis (CSTH), and the International Society of Thrombosis and Hemostasis (ISTH) scoring system have all been used to diagnose sepsis-induced coagulopathy (SIC) [9,10].

Meanwhile, the Can Rapid risk stratification of Unstable angina patients Suppress adverse outcomes with Early Implementation of the ACC/AHA Guidelines (CRUSADE) score is also a powerful score for the diagnosis of blood coagulation status changes and prognosis in multiple emergency and critical conditions [11,12]. At present, the above score is mainly used for the evaluation and diagnosis of SIC. Still, whether it can accurately predict the prognosis of SIC patients is unclear, and related reports are also lacking. We investigated the predictive value of the above four scoring systems for predicting SIC patients using data from our institution’s SIC cohort, and the findings are summarized below.

## 2 Materials and methods

### 2.1 Materials

This is a retrospective study; patients with SIC who were hospitalized in our hospital’s intensive care unit between August 2021 and August 2022 were included in the research. According to their prognosis 14 days after admission [13], they were split into two groups: those with a bad prognosis (*n* = 35) and those with a favorable prognosis (*n* = 57). The criteria for poor prognosis were death due to all causes or still staying in the ICU 14 days after admission.

Inclusion criteria: (1) age greater than 18 years old, (2) sepsis diagnosis conforms to sepsis 3.0 standard [14], (3) SIC diagnosis conforms to 2019 ISTH diagnostic standard [9], and (4) relevant laboratory data are complete.

Exclusion criteria: (1) those who give up treatment, (2) coagulation disease was complicated at admission, (3) those who are complicated with malignant tumors, and (4) the length of stay in ICU is less than 24 h. The research related to human use has complied with all the relevant national/regulations. The Ethics Committee of 3201 Hospital has approved institutional/policies and accordance with the Helsinki Declaration’s tenets.


**Informed consent:** Informed consent has been obtained from all individuals included in this study.
**Ethical approval:** The research related to human use has been complied with all the relevant national regulations, institutional policies and in accordance with the tenets of the Helsinki Declaration, and has been approved by the authors’ institutional review board or equivalent committee.

### 2.2 Methods

#### 2.2.1 General data

From the electronic medical record system, we gathered the patients’ sex, ages, body mass indexes (BMIs), complications (such as diabetes, chronic renal disease, hypertension, COPD, initial antibiotic application, and the need for mechanical ventilation), and APACHE II scores at admission.

#### 2.2.2 Statistics of four scores

(1) JAAM includes platelet (PLT), prothrombin time (PT), fasting blood glucose (FBG), fibrinogen degradation products, and systemic inflammatory response syndrome (SIRS) scores. The higher the score the greater the risk of abnormal coagulation function. (2) ISTH includes PLT, PT, FBG, and D-dimer. The higher the score the greater the risk of abnormal blood coagulation function. (3) CDSS includes PLT, fibrinogen (FIB), D-dimer, activated partial thromboplastin time (APTT), PT, and disseminated intravascular coagulation (DIC)-related primary diseases and clinical manifestations. CDSS scoring rules: there is a primary disease that causes DIC (yes = 2, no = 0); severe or multiple bleeding tendencies that cannot be explained by primary disease (yes = 1, no = 0); microcirculatory disturbance or shock that cannot be explained by primary disease (yes = 1, no = 0); extensive skin and mucosal embolism, focal ischemic necrosis, shedding and ulceration, unexplained lung, kidney, brain, and another organ failure (yes = 1, no = 0); the platelet count in non-malignant hematological diseases (>100 × 10^9^/L = 0, 80–100 × 10^9^/L = 1, <80 × 10^9^/L = 2); platelet count (<50 × 10^9^/L = 1); d-dimer (<5 mg/L = 0, 5–9 mg/L = 2, >9 mg/L = 3); pT and APTT prolongation (PT prolongation <3 s and APTT prolongation <10 s = 0, PT prolongation >3 s or APTT prolongation 6 s = 2), FIB (>1.0 g/L = 0, ≤1.0 g/L = 1). (4) CRUSADE score includes eight indicators: gender, baseline hematocrit, creatinine clearance, systolic blood pressure, heart failure, history of vascular disease or stroke, history of diabetes, and heart rate (total score 1–91 points). The higher the score the greater the risk of abnormal coagulation function.

### 2.3 Statistical analysis

SPSS21.0 was used to evaluate the gathered experimental data. The *t*-test was used to compare the experimental data where the measurements followed a normal distribution (represented by *X* ± *S*), and the *χ* ^2^ test was used to compare the counting data (represented by several instances or rates). The correlations were analyzed using the Spearman technique. The prognosis of SIC patients was studied using multivariate logistic regression. Using a receiver operating characteristic curve, we compared the scores’ ability to predict SIC patients’ outcomes. We ran the same tests on the data from both sides and found that the difference was statistically significant at the *p* < 0.05 level.

## 3 Results

### 3.1 Group data comparison

Gender, age, BMI, comorbidities, and need for mechanical ventilation were not significantly different between the two groups (all *p* > 0.05). However, the APACHE II score of those with a poor prognosis at admission was greater than that of those with a favorable prognosis (*p* < 0.05) (Table 1).

**Table 1:** Comparison of general data between two groups

Item	Poor prognosis group (*n* = 35)	Good prognosis group (*n* = 57)	*t*/*χ* ^2^ value	*p* Value
Gender (male)	20	35	0.043	0.836
Age (years)	58.64 ± 10.36	60.13 ± 11.64	0.621	0.536
BMI (kg/m^2^)	23.66 ± 4.36	24.10 ± 4.53	0.459	0.648
**Complication**				
Diabetes	13	13	2.198	0.138
Hypertension	10	11	1.059	0.304
Chronic nephrosis	6	8	0.162	0.687
COPD	4	6	0.018	0.893
Mechanical ventilation (cases)	28	45	0.015	0.904
APACHE II at admission (min)	17.46 ± 4.31	15.64 ± 3.88	2.094	0.039
**Initial antibiotic application**				
Reasonable	31	52	0.173	0.677
Unreasonable	4	5		

### 3.2 Comparison of four scores and coagulation function indexes in SIC patients with different prognoses

When comparing the two groups, those with a bad prognosis had higher JAAM, ISTH, CDSS, and CRUSADE scores (all *p* < 0.05), whereas those with a favorable prognosis had higher FIB, D-dimer, APTT, and PT levels (both *p* > 0.05) (Table 2).

**Table 2:** Comparison of four scores and coagulation function indexes in SIC patients with different prognosis

Item	Poor prognosis group (*n* = 35)	Good prognosis group (*n* = 57)	*T* value	*p* Value
JAAM (points)	6.13 ± 1.58	5.39 ± 1.36	2.381	0.019
ISTH (points)	6.93 ± 1.44	6.01 ± 1.26	3.219	0.002
CDSS (points)	7.16 ± 1.34	5.83 ± 1.16	5.031	<0.001
CRUSADE (points)	34.36 ± 10.63	28.64 ± 9.71	2.646	0.010
PT/s	18.35 ± 4.53	16.97 ± 4.46	1.432	0.156
APTT (s)	70.66 ± 43.53	61.56 ± 40.53	1.016	0.312
FIB (g/L)	3.64 ± 1.50	3.49 ± 1.44	0.477	0.634
D-dimer (mg/L)	12.66 ± 5.33	10.63 ± 4.73	1.904	0.060

### 3.3 Correlation analysis of four scores with coagulation function in SIC patients

For both JAAM and ISTH (all *p* > 0.05 except D-dimer, *p* < 0.05); CDSS (all *p* < 0.05 except FIB, *p* > 0.050); CRUSADE (all *p* < 0.05) (Table 3).

**Table 3:** Correlation analysis of four scores with coagulation function in SIC patients

	JAAM		ISTH		CDSS		CRUSADE	
	*r*	*p*	*r*	*p*	*r*	*p*	*r*	*p*
D-dimer	0.253	0.016	0.335	<0.001	0.401	<0.001	0.377	<0.001
FIB	0.165	0.154	0.149	0.135	0.167	0.069	0.264	0.015
PT	0.036	0.536	0.185	0.443	0.351	0.020	0.293	0.003
APTT	−0.134	0.559	0.165	0.315	0.256	0.013	0.216	0.006

### 3.4 Prognostic variables in individuals with SIC: a multivariate analysis

The prognosis of SIC patients 14 days after hospitalization was used as the dependent variable, and the APACHE II, JAAM, ISTH, CDSS, and CRUSADE scores upon admission that were significant in univariate analysis were used as the independent factors. All of the independent variables were continually assigned to actual values. Patients with SIC with a higher JAAM, ISTH, CDSS, CRUSADE, or APACHE II score at admission had a better prognosis than those with lower scores (all *p* < 0.05, Table 4).

**Table 4:** Multivariate evaluations of prognostic variables in SIC patients

Risk factor	*B* value	SE value	Ward value	OR value	95% CI	*p* Value
JAAM	1.052	0.373	7.958	2.864	1.379–5.949	0.026
ISTH	0.183	0.379	9.742	3.264	1.553–6.861	0.023
CDSS	1.352	0.385	12.341	3.867	1.818–8.224	<0.001
CRUSADE	1.268	0.382	11.015	3.553	1.680–7.512	0.010
APACHE II at admission	0.951	0.370	6.605	2.588	1.253–5.345	0.033

### 3.5 Receiver operating characteristic (ROC) analysis of the prognostic value of four scores in patients with SIC

**Figure 1:**
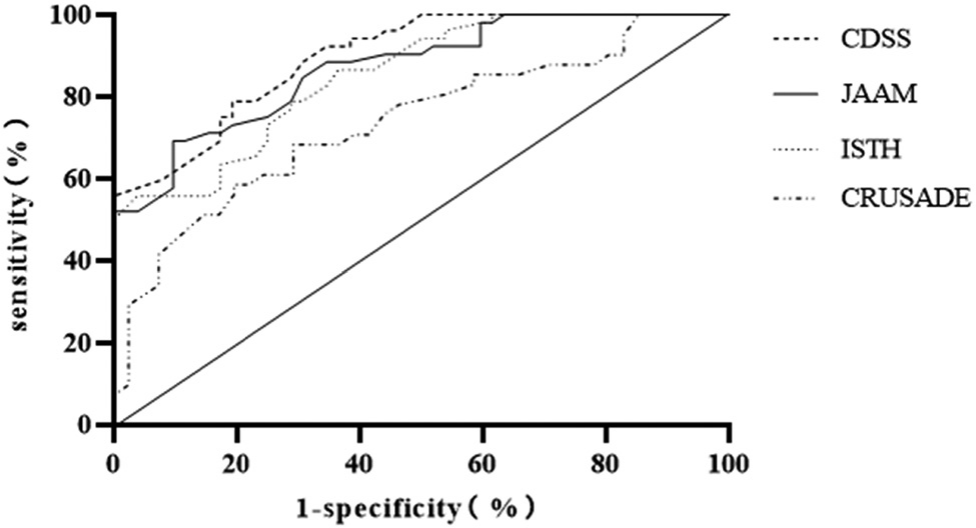
ROC analysis results.

The area under the prognosis curve (AUC) for predicting SIC patients using the JAAM, ISTH, CDSS, and CRUSADE scores was 0.896, 0.870, 0.852, and 0.737, respectively, with 95% CI being 0.840–0.952, 0.805–0.936, 0.783–922, and 0.629–0.845, respectively (all *p* < 0.05, Table 5 and Figure 1).

**Table 5:** ROC analysis of the predictive value of four scores in patients with SIC

Indicator	AUC	Sensitivity (%)	Specificity (%)	95% CI	Standard error	*p* Value
JAAM	0.896	84.95	82.66	0.840–0.952	0.029	<0.001
ISTH	0.870	82.66	81.86	0.805–0.936	0.033	<0.001
CDSS	0.852	81.85	80.25	0.783–0.922	0.035	<0.001
CRUSADE	0.737	70.75	68.65	0.629–0.845	0.055	<0.001

## 4 Discussion

SIC is a life-threatening condition characterized by abnormal clotting and bleeding in patients with sepsis. The body releases many endotoxin and inflammatory cytokines when sepsis occurs, directly damaging vascular endothelial cells. Endothelial cell injury can lead to excessive activation of the inflammation–coagulation pathway, leading to coagulation dysfunction [15,16,17,18,19]. Meanwhile, the inflammatory reaction leads to endothelial cell swelling and gap enlargement, leading to increased capillary permeability and thus to capillary leakage, which aggravates the insufficient perfusion of tissues and organs, and finally leads to multiple organ dysfunction. Accurate assessment of the severity and prognosis of SIC is crucial for appropriate management and clinical decision-making. Various scoring systems have been developed to evaluate coagulation abnormalities in critical illness, including the ISTH, the CSTH, the JAAM, and the CRUSADE. This study aims to compare the predictive value of these scoring systems in patients with SIC. Hence, the combined evaluation of coagulation function markers is helpful for the early detection of SIC [20,21,22,23].

Our study examined the association between various factors and patient prognosis in a cohort of individuals with a specific medical condition. We found that gender, age, BMI, comorbidities, and the need for mechanical ventilation did not show a significant difference between the two groups, suggesting that these factors may not be strong predictors of prognosis in this population (all *p* > 0.05). However, we did observe that the APACHE II score, a widely used severity scoring system, was significantly higher in patients with a poor prognosis compared to those with a favorable prognosis (*p* < 0.05), indicating its potential utility as a prognostic indicator. These findings emphasize the importance of considering comprehensive assessment tools, such as the APACHE II score, to evaluate patient outcomes accurately and guide clinical decision-making.

The ISTH scoring system, also known as the ISTH overt DIC score, is widely used for diagnosing and prognosis DIC. It incorporates clinical and laboratory parameters such as platelet count, FIB level, PT, and fibrin degradation products [24,25]. Our results presented in Table 2 demonstrate that the group of patients with a poor prognosis had significantly higher ISTH scores and Coagulation Disorders Severity Scale (CDSS) scores (all *p* < 0.05) compared to the group with a favorable prognosis.

These findings suggest that higher ISTH and CDSS scores may indicate a more severe coagulopathy and are associated with an unfavorable prognosis. In contrast, the group with a favorable prognosis exhibited higher levels of FIB, D-dimer, APTT, and PT (both *p* > 0.05). However, the differences were not statistically significant. These findings suggest that higher FIB, D-dimer, APTT, and PT levels may be associated with a more favorable prognosis, but further investigation is required to establish a significant correlation.

Several studies have evaluated the predictive value of the ISTH score in patients with SIC. SIC, which is easy to calculate and consistent with the pathophysiology of fibrinolytic inhibition and high organ dysfunction in sepsis, has been proven to be controversial [26]. For instance, Chen et al. study revealed that 306 (66.7%) of 452 patients (mean age, 65 years) were men, and the median SOFA and APACHE II scores were 6 and 15, respectively. Around 132 patients (29.2%) died in 28 days; 12.2% had ISTH overt-DIC, 44.7% had JAAM, and 25.4% had positive SIC. SIC and ISTH overt-DIC both discriminated 28-day all-cause mortality well. However, the SIC had a greater calibration curve coincidence for 28-day all-cause mortality than ISTH overt-DIC. JAAM DIC did not independently predict 28-day all-cause death in sepsis (RR, 1.115, [95% CI 0.660−1.182], *p* = 0.684) (Chen et al., 2023).

Another study by Iba et al. [10] found that the ISTH score predicted organ dysfunction and mortality in patients with SIC. Initially, 149 of 151 ISTH-diagnosed overt DIC patients had SIC. About 93.9% of the 49 patients with overt DIC had SIC between days 2 and 4. Baseline SIC predicted death with 86.8% sensitivity, compared to 64.5% for overt DIC (*p* < 0.001). On days 2, 4, and 7, SIC had a statistically significantly higher sensitivity than overt DIC (96.1, 92.3, and 84.4 vs 67.1, 57.7, and 50.0%). SIC’s specificity was consistently lower [10].

A similar study by Iba et al. [4,7,9] demonstrated that higher ISTH scores were associated with increased mortality in patients with sepsis. Using the m-JAAM, s-JSTH, SCI, and ISTH criteria, the AUC for predicting in-hospital 28-day mortality was 0.745, 0.763, 0.760, and 0.730, respectively. Patients who met the m-JAAM and SIC criteria were more likely to do so than those who completed the s-JSTH and ISTH criteria (43.2, 56.1 vs 25.0, 22.6%, *p* < 0.05) [4,7,9].

The CSTH scoring system, developed by the CSTH, focuses on evaluating the severity of coagulation disorders in critically ill patients. It incorporates platelet count, PT, FIB level, and D-dimer. Several studies have investigated the predictive value of the CSTH score in patients with SIC. For example, a study by Wang et al. [6] supported these findings by demonstrating that elevated CSTH scores were associated with a higher risk of organ dysfunction and mortality in patients with SIC. These studies suggest that the CSTH score has the potential as a reliable predictive tool for assessing SIC severity and clinical outcomes. Another study by Xu et al. [27] demonstrated that the CSTH score was an independent predictor of mortality in septic patients with coagulation dysfunction.

The JAAM criteria include the SIRS score and PLT reduction ratio and are recommended for early diagnosis of sepsis DIC and guidance. Our finding JAAM levels were substantially more significant in the group with a poor prognosis (*p*-value <0.05). Poor and favorable prognosis groups had similar FIB, D-dimer, APTT, and PT levels (*p* > 0.05). A multivariate logistic regression analysis showed that the entrance scores of JAAM, ISTH, CDSS, CRUSADE, and APACHE II were all independently associated with the prognosis of SIC patients (with a *p*-value less than 0.05 for all).

Several studies have examined the predictive value of the JAAM score in patients with SIC. For instance, a study by Jhang and Park [1] demonstrated that higher JAAM scores were associated with increased mortality in patients with sepsis and DIC. SIC identified 84.4% of 135 coagulopathy cases. The modified SIC score – pSOFA, PT, and D-dimer – diagnosed SIC in 68 (50.4%) patients. pSOFA, DIC, and SIC values linked (*p* < 0.001) 28-day mortality was 18.7% in coagulopathy harmed patients. Modified SIC scores independently predicted 28-day mortality. The modified SIC score predicted 28-day mortality better than SIC and ISTH DIC (*p* < 0.05) [1].

Another study by Gando et al. [28] demonstrated that 141 JAAM DIC patients (44.9%) differed from non-DIC patients in significant bleeding, multiple organ dysfunction syndrome (MODS), and outcome. Maximum JAAM DIC scores independently predicted patient death in stepwise logistic regression. Early JAAM DIC criteria recognized all ISTH overt DIC cases. ISTH overt DIC patients had higher death, severe bleeding, and MODS rates than JAAM DIC patients. The ISTH and JAAM overt DIC scores and incidence increased stepwise. DIC patients with trauma and sepsis had different MODS and Sequential Organ Failure Assessment ratings. Mortality rates were the same [28]. Additionally, Wada et al. [29] found that the JAAM score effectively predicted the development of MODS and mortality in patients with SIC. These findings highlight the potential of the JAAM score as a valuable prognostic tool for assessing the severity and clinical outcomes of SIC [29].

Although the CRUSADE scoring system was primarily developed for risk stratification in patients with unstable angina, its prognostic value in SIC has been explored in limited studies [30]. However, no specific studies comparing the predictive value of CRUSADE with the other scoring systems in patients with SIC were identified in the literature. A single survey by Quteineh et al. [31] reported that an 89-year-old man with sepsis, diffuse intravascular coagulopathy, acute renal damage, and upper GI bleeding arrived at the emergency department. He was treated for septic shock, diabetic ketoacidosis, and coagulopathy in the ICU and sent home on medicines [31]. According to our findings, the CRUSADE score can effectively predict the incidence of coagulation dysfunction and bleeding events in patients with toxemia during hospitalization. The high-risk and highly high-risk patients with CRUSADE scores can effectively reduce the occurrence of DIC in sepsis at admission. In addition, in this study, it was found that the levels of CRUSADE were significantly higher in the group of patients with a poor prognosis compared to those with a good prognosis (with a *p*-value of less than 0.05). No significant difference was found between the poor and favorable prognosis groups with FIB, D-dimer, APTT, and PT levels (*p* > 0.05). Further research is warranted to determine the utility and comparative effectiveness of the CRUSADE score in predicting outcomes in this specific patient population.

The results show that the SIC tools JAAM, ISTH, CDSS, and CRUSADE, validated by large samples, have clinical importance in predicting patient outcomes. Conventional measures of coagulation function, such as FIB, D-dimer, APTT, and PT, were less accurate in representing patient prognosis. The reason may be that while the coagulation status of SIC patients is dynamic and complicated, FIB, D-dimer, APTT, and PT can only reflect a part of the characteristics of coagulation function changes [32] rather than reflecting the overall picture of abnormal coagulation function in patients with sepsis.

## 5 Conclusions

In conclusion, the prognosis of SIC patients may be predicted to some degree using the JAAM, ISTH, CDSS, and CRUSADE scores, with the CDSS score being the most accurate. This research has important practical implications for improving care for people with SIC. Since this study only looked at one location and a small number of patients, the conclusion needs to be reinforced and supplemented by data from a broader clinical sample. Overall, the ISTH, CSTH, and JAAM scoring systems have shown consistent prognostic value in assessing the severity and predicting clinical outcomes in patients with SIC. These scoring systems incorporate various clinical and laboratory parameters to provide a comprehensive evaluation of coagulation abnormalities. However, further research is needed to validate and directly compare the prognostic accuracy of these scoring systems in large, multicenter studies.
